# TRIPOD+AI statement: updated guidance for reporting clinical prediction models that use regression or machine learning methods

**DOI:** 10.1136/bmj.q902

**Published:** 2024-04-18

In this paper by Collins and colleagues (*BMJ* 2024;385:e078378, doi:10.1136/bmj-2023-078378, published 16 April 2024), minor changes have been made to correct the authors’ affiliations. The article and PDF have been updated.

